# Will Host Genetics Affect the Response to SARS-CoV-2 Vaccines? Historical Precedents

**DOI:** 10.3389/fmed.2022.802312

**Published:** 2022-03-11

**Authors:** Maria K. Smatti, Hebah A. Alkhatib, Asmaa A. Al Thani, Hadi M. Yassine

**Affiliations:** ^1^College of Health and Life Sciences, Hamad Bin Khalifa University, Doha, Qatar; ^2^Biomedical Research Center, Qatar University, Doha, Qatar

**Keywords:** SARS-CoV-2, COVID-19, vaccines, SNPs, host genetics

## Abstract

Recent progress in genomics and bioinformatics technologies have allowed for the emergence of immunogenomics field. This intersection of immunology and genetics has broadened our understanding of how the immune system responds to infection and vaccination. While the immunogenetic basis of the huge clinical variability in response to the severe acute respiratory syndrome coronavirus 2 (SARS-CoV-2) infection is currently being extensively studied, the host genetic determinants of SARS-CoV-2 vaccines remain largely unknown. Previous reports evidenced that vaccines may not protect all populations or individuals equally, due to multiple host- and vaccine-specific factors. Several studies on vaccine response to measles, rubella, hepatitis B, smallpox, and influenza highlighted the contribution of genetic mutations or polymorphisms in modulating the innate and adaptive immunity following vaccination. Specifically, genetic variants in genes encoding virus receptors, antigen presentation, cytokine production, or related to immune cells activation and differentiation could influence how an individual responds to vaccination. Although such knowledge could be utilized to generate personalized vaccine strategies to optimize the vaccine response, studies in this filed are still scarce. Here, we briefly summarize the scientific literature related to the immunogenetic determinants of vaccine-induced immunity, highlighting the possible role of host genetics in response to SARS-CoV-2 vaccines as well.

## Introduction

Vaccination has become one of the most effective public health strategies to prevent infectious diseases in the modern medicine. Undeniably, it has saved millions of lives by reducing the burden of many serious infections such as polio, tuberculosis, measles, and tetanus. Currently, the entire world is in a battle against SARS-CoV-2, which emerged at the end of 2019 and caused the coronavirus disease 19 (COVID-19). The virus has affected almost 400 million people and has claimed over 5 million lives worldwide (1). Yet, there is no decisive therapy to treat SARS-CoV-2 infection until now, and therefore, vaccines are considered the only hope to control the spread of the virus.

Despite the great success of vaccines throughout the history, the field of vaccinology is still dominated by the traditional empiric model of “isolate-inactivate-inject,” which translates into a population-level model of “same dose for everyone for every disease” (2). Clearly, this approach is limited by the incomplete knowledge on immunogenetic determinants of vaccine effectiveness as well as the population and individual heterogeneity in vaccine-induced immunity. Therefore, the poor immune response in some individuals to vaccines remains unexplained.

Population based studies highlighted the relatively high percentages of vaccine failure and the possible role of genetic factors in that. It was found that ~2–10% of individuals receiving the measles vaccine fail to produce protective immunity (3). Also, vaccination against rubella indicated that 2–5% of vaccinated individuals do not seroconvert. Not only that, but also those who respond to the vaccine showed a great variability in the immune response, which is believed to be heritable (4). Moreover, Hepatitis B vaccine failure was estimated to be 5–10% (5). Ganczak et al. reported an association between the homozygous genotype of CCR5Δ32 of the *CCR5* gene and reduced HBV vaccine immunogenicity (6). This genetic mutation exhibits a characteristic ethnical distribution, being more frequent in Europeans, and thus, may influence their response to the HBV vaccine. Inter-individual differences in response to Anthrax Vaccine Adsorbed (AVA) had also suggested the potential host genetic influences, as evidenced by the observed variability in the protective antigen-specific antibodies level between Europeans and African-Americans (7). Furthermore, genetic polymorphisms of the HLA, cytokines, innate immunity and viral receptor, and other genes, were found to account for almost 30% of the inter-individual variation in measles vaccine-specific humoral immunity (8).

It is now well-acknowledged that an individualized medicine approach mandates the integration of the mechanistic understanding of all the factors that could contribute to vaccine effectiveness, including host immunogenomics. This, in turn, aims to provide the right vaccine to the right patient, with the right reason, at the right dose (2). Although researches had begun looking into the host genetics, aiming to find immunogenomic clues to vaccine-response and factors behind vaccine failure, investigations in this field are still very limited.

The paradigm of personalized medicine has been applied in the current SARS-CoV-2 in an effort to understand the large clinical variability observed between individuals as well as populations. While several large-scale studies highlighted the crucial role of genetic diversity in response to COVID-19, the contribution of host genetics in response to SARS-CoV-2 vaccines is unknown. Importantly, the need for personalized approaches could be more crucial for SARS-CoV-2 vaccines compared to other vaccines. The reason behind this is the large inter-individual differences that was reported in response to SARS-CoV-2 infection, where host genetics factors showed to contribute to SARS-CoV-2 clinical variability and modulate response to infection. This variability could also be translated into vaccine responsiveness. Moreover, the global spread of SARS-CoV-2 pandemic, which in turn, led to the wide administration of SARS-CoV-2 vaccines, could increase the chance of low vaccine efficacy or high risk of adverse reactions at certain populations or individuals. Hence, it is significant to understand the immunogenetic factors underlying SARS-CoV-2 vaccine effectiveness and adverse responses at both individual and population levels.

Here, we review the role of genetics in response to vaccination to other pathogens, aiming to draw attention to this important field, especially that SARS-CoV-2 vaccines are currently being distributed and evaluated.

## Overview on the Immune response to Viral Infections

It is well-known that immune responses to viral infections involve all arms of the immune system. This begins with pathogen recognition and antigen presentation and is then followed by a cascade of immune defense mechanisms of innate and adaptive immunity. The innate immune system is the first line of defense. It is triggered by encountering damage-associated molecular patterns (DAMPs) released from infected tissue or dead cells or pathogen-associated molecular patterns (PAMPs), such as viral RNA and DNA (9). Virally induced DAMPs and PAMPs stimulate tissue-resident macrophages and activate multiple innate immune pathways through Toll-Like receptors (TLRs), NLRP3/inflammasome activation, or by triggering cytoplasmic DNA sensors such as cGAS-STING and RIG-I-MAVS. This, in turn, derives the production of pro-inflammatory cytokines and chemokines, which subsequently leads to the stimulation of antiviral gene expression and the recruitment of more innate and adaptive immune cells for viral control and tissue hemostasis. The production of type I and type III interferons (IFNs) as a part of innate immunity initiates intracellular antiviral defense pathways while the release of IL-6 and IL-1β stimulates the recruitment of neutrophils and cytotoxic T cells (10). Paradoxically, the dysregulated inflammatory cascade initiated by macrophages could contribute to tissue damage leading to cytokine storm as previously reported from different viral infections, including SARS-CoV-2 (9).

Following and complementing the innate immune response, the adaptive immune system responds to pathogens by producing pathogen-specific humoral and cellular immunity, with T and B cells acting as the key players. T-cell mediated immune response represents an essential arm in mediating adaptive immunity to a variety of pathogens. Pathogen peptides presented by the MHC complexes on the surface of antigen-presenting cells (APCs), such as dendritic cells (DCs), stimulate the activation, proliferation, and differentiation of naïve CD8^+^ and CD4^+^ T-cells. Subsequently, these cells undergo clonal expansion by interleukin-2 (IL-2), and differentiate into effector T cells in the presence of a set of cytokines engaging and activating their respective cytokine receptors (11, 12). Importantly, achieving an effective viral clearance requires CD8^+^ effector T cell-mediated killing of infected cells in addition to CD4^+^ T cell-mediated enhancement of CD8^+^ and B cell responses.

On the other hand, humoral immunity, particularly the production of neutralizing antibodies, is of a central importance in combating viral infections. It is evidenced that T-independent B cell response contribute substantially to highly stable antibody repertoires, providing humoral barriers to protect against invading pathogens. However, producing humoral memory through long-lived plasma cells that elicit specific antibodies of adapted avidity and function is T-cell dependent (13). Taken together, an efficient immunological memory is achieved by the collective involvement of both T and B cells responses.

## Overview on the Immune Response to Vaccination

The innate immune system can sense vaccines through the pattern-recognition receptors (PRRs), such as TLRs. For instance, the influenza virus live-attenuated vaccine activates plasmacytoid DCs (pDCs) *via* TLR7 (14). Another example is the yellow fever vaccine (YF-17D), which stimulates multiple TLRs on DCs, including TLR2, TLR3, TLR7, TLR8, and TLR9 (15). Importantly, it was shown that deficiency in any TLR substantially impaired the cytokine production in mice model (15). Vaccines based on synthetic nanoparticles containing TLR ligand have also shown to induce a synergistic enhancement of both the affinity of neutralizing antibodies as well as specialized T-cell responses (16). Most importantly, polymorphisms in TLR genes have been previously linked to immune response following vaccination. For example, variants in the *TLR3* gene and its associated signaling genes were associated with low measles antibody and lymphoproliferative immune responses in vaccinated individuals (17). This highlights the central role of TLRs in vaccine-induced innate and adaptive immunity.

Most vaccines are believed to confer protection by inducing B-cells mediated immunity that results in antibody production, although they can induce T cell responses as well. Polysaccharide vaccines, particularly, are completely T-cell independent, in contrast to vaccines based on proteins combined with polysaccharides, which can induce B and T cell responses (18). Recently, there has been an increasing interest in understanding the role of T cells in vaccine-induced protection; especially that antibodies level is not the only indicator of vaccine effectiveness. The main goal of any T-cell-based vaccine is to induce antigen-specific memory T cells. Following vaccination, naïve CD4+ T cells differentiate to functionally distinct populations of helper T cells (Th1, Th2, Th17, Th21, T follicular helper, Th22, or Th9), which are involved in different defense mechanisms. On the other hand, naïve CD8+ T cells can differentiate into effector cells, while memory T cells reside as precursor cells in lymphoid organs and differentiate rapidly to effector cells upon stimulation (14). New vaccine platforms such as lipid nanoparticles (LNP) based vaccines induce T cells responses that depend on the DC subsets and PRRs involved. For instance, mRNA-LNP vaccines have been shown to induce Th1 and T follicular helper cells (Tfh), most probably through the engagement of TLRs (19). Adenovirus vectors, on the other hand, are considered one of the most potent vaccines in inducing CD8+ T cell responses in addition to sustained B and CD4+ T cell responses (20). However, the absence of individual TLRs does not seem to affect antigen-specific CD8+ T cell responses elicited by adenovirus vectors, suggesting that this type of vaccine involves multiple redundant MyD88 (TLR adapter protein)-dependent signaling pathways (14).

## Heterogeneity in Vaccine-Induced Immune Response

**Figure 1:**
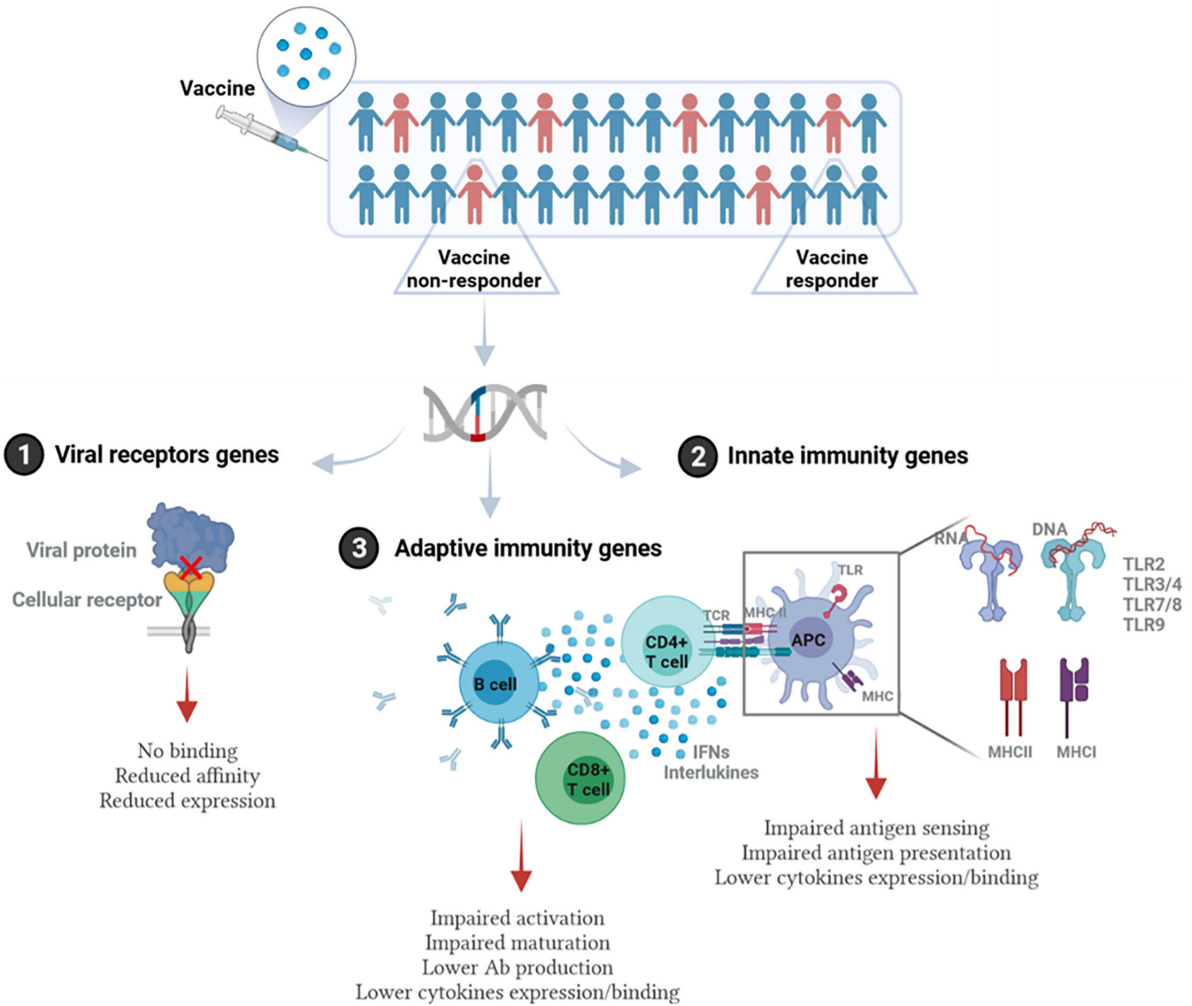
Immunogenetic pathways involved in vaccine response. Individuals/populations with lower vaccine efficacy could carry genetic polymorphisms in: (1) Genes encoding viral receptors on the host cells. This could affect the binding affinity of viral antigen and cellular receptor, virus entry, or the level of receptor expression. (2) Genes related to the innate immunity. This includes genes encoding pathogen recognition receptors (PRRs) such as different types of TLRs, and MHC (HLA) genes that are essential for antigen presentation, as well as genes encoding cytokines and cytokine receptors. (3) Genes related to adaptive immune response such as T and B cell receptors, genes related to activation or differentiation of adaptive immune cells, and antibody production. This figure was generated using Biorender.

The influence of host genetics on vaccine response occurs if polymorphisms or mutations exist in genes related directly or indirectly to the host immune response to the vaccine. This involves but is not limited to genes related to cellular receptors of viral proteins/adjuvants, antigen presentation, innate immunity (such as TLRs), signaling molecules, cytokine genes, cytokine receptor genes, HLA, immunoglobulin Gm and Km allotypes, vitamin A and D receptor genes, and many other genes (21). Figure 1 illustrates the main pathways where genetic polymorphisms could modulate response to vaccination.

### Twin Studies

A considerable clue for the influence of genetics on vaccine- and natural-induced immunity comes from twin studies. These studies represented a pivotal model to differentiate genetics from environmental and other factors affecting immune response phenotypes. Heritability, which is estimated as the ratio of genetic variance to total variance within pairs, was used to assess genetics-vaccines associations (21). Using this approach, very early studies pinpointed the heritability to measles-mumps-rubella-II (MMRII) vaccine response. For instance, through examining the antibody level in 100 healthy twins who received MMRII vaccine, a study found that heritability to measles almost reached 90%, while heritability to rubella and mumps was 46 and 39%, respectively (22). Similarly, other reports evidenced the heritability of vaccine-induced antibody response to hepatitis viruses, ranging from 60% for recombinant hepatitis B surface antigen (HBsAg) vaccine, to 36% in the inactivated hepatitis A vaccine (23). Of note, only 40% of this heritability pattern was explained by HLA genes, compared to non-HLA genes, which contributed to 60% of the cases. This underscores the importance of exploring genetic polymorphisms with a broad prospect and at the whole genome level in order to better identify genetic factors contributing to vaccine responsiveness. Additional twin studies had confirmed the dominant role of non-HLA genes in the humoral response to vaccination to hepatitis B, oral polio, tetanus, and diphtheria, which all had high heritabilities (77, 60, 44, and 49%, respectively). In addition to the antibody response, interferon-γ and interleukin-13 responses also showed a high degree of heritability to some BCG vaccine antigens (39–65%). Yet, these responses were mainly modulated by HLA class II genes (24). Taken together, these studies provided a glimpse on the importance of gene variation in the modulating the humoral immune response to different vaccines, and opened the door for a more comprehensive research in this field.

### Genome-Wide Association Studies

In recent years, the advancements in genomics and bioinformatics have paved the way for implementing genome-wide association studies (GWAS) to investigate the link between host genetics and response to vaccines. Several GWAS have discovered single nucleotide polymorphisms (SNPs) in genes related to the innate and adaptive immune responses. However, despite the continuously growing number of vaccine-associated GWASs, these studies are either clustered within specific ethnic groups, or focused on a limited number of pathogens. Most of the currently available reports are on vaccine response to hepatitis B, measles, rubella, influenza A, smallpox, anthrax, and mumps (4, 5, 7, 25–29).

**Figure 2:**
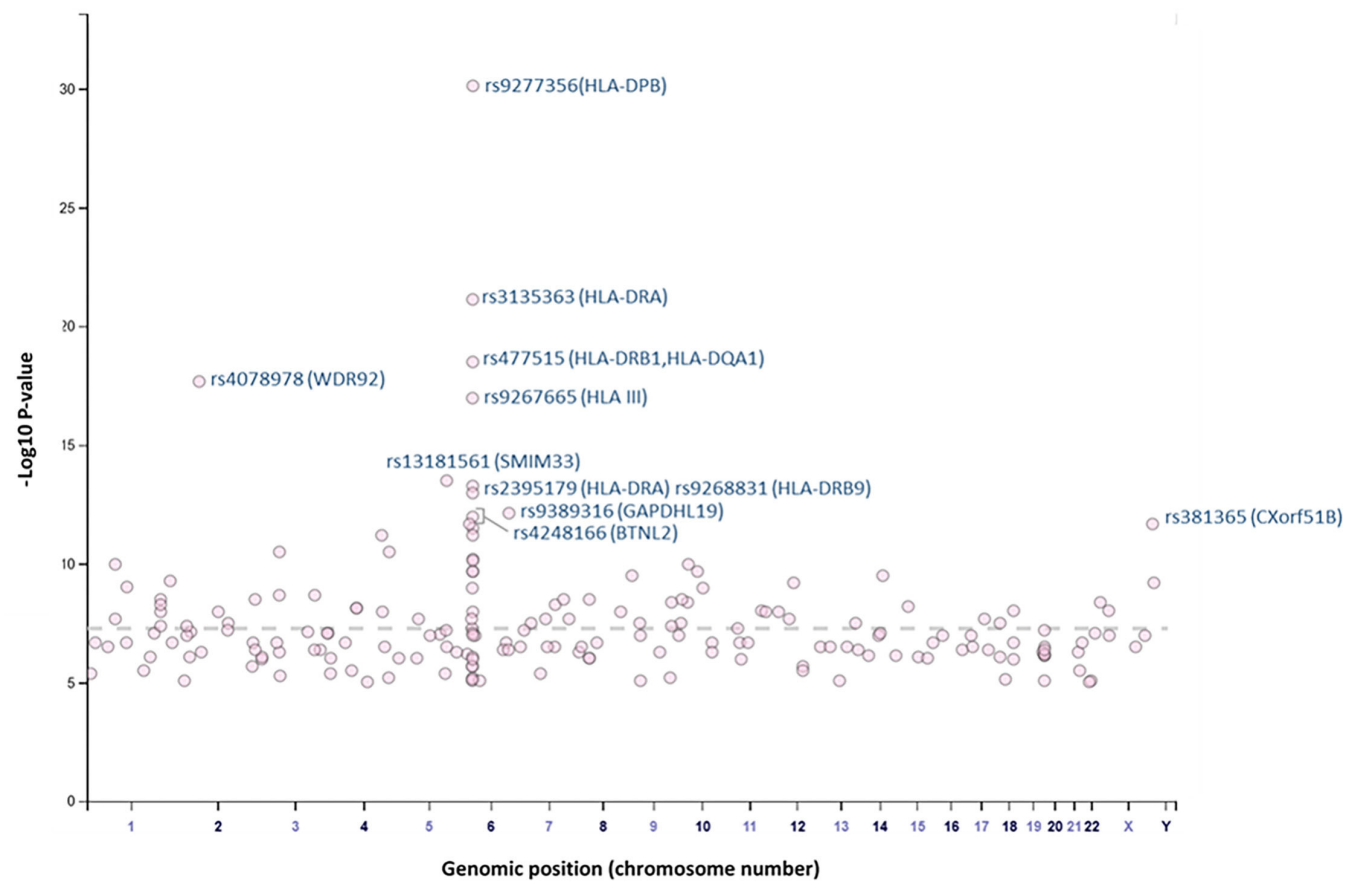
Plot of GWAS Catalog associations for vaccine response related SNPs. The data and plot were retrieved from the GWAS catalog, an open database. All associations with “response to vaccine” phenotype are plotted. The top 10 SNPs are labeled with the rs identifiers.

**Figure 3:**
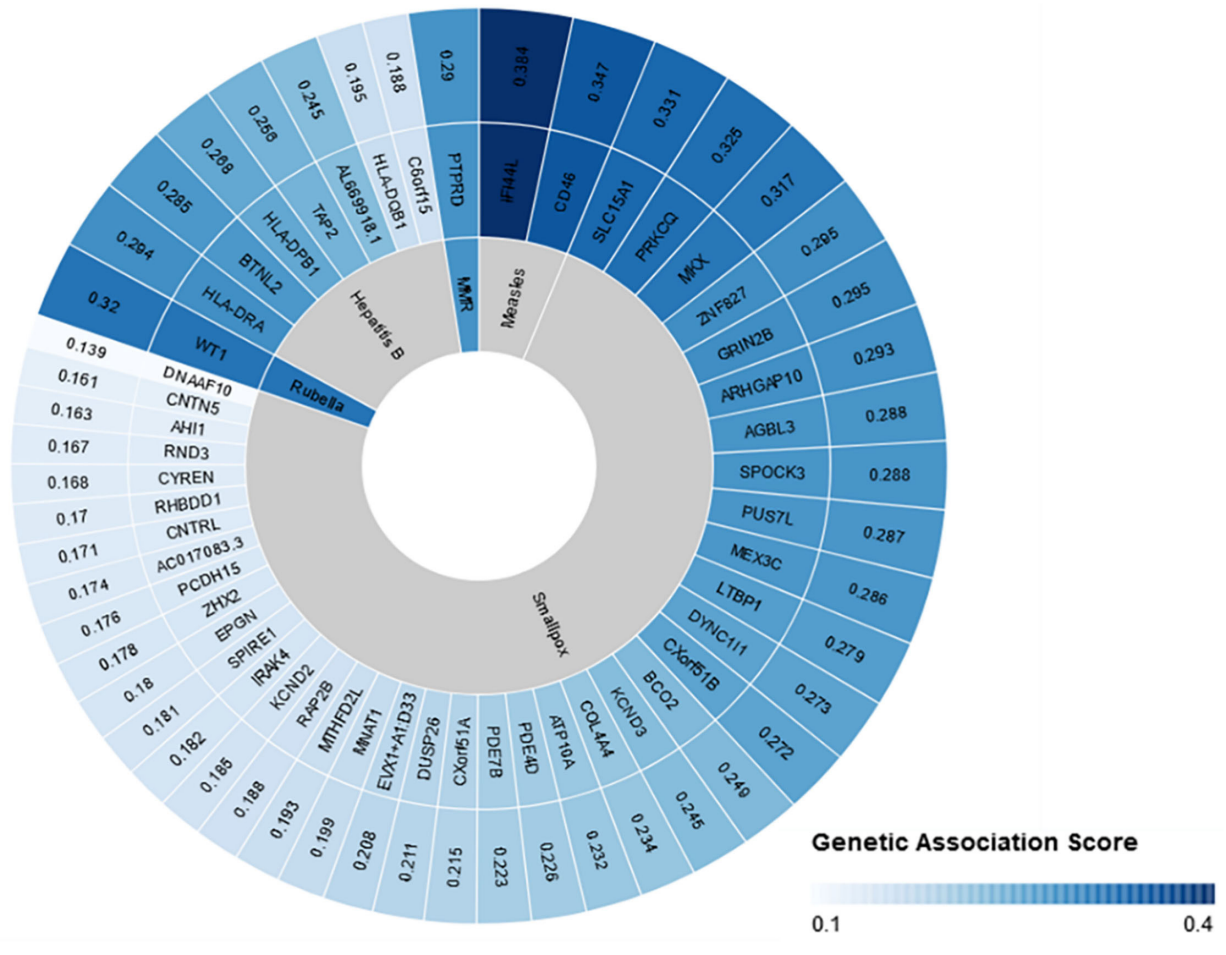
Open Targets genetics association scores for genes linked to vaccine response. Open target platform was used to search for all the associations under “response to vaccine” phenotype. The top 50 associations are plotted with the association score, the name of the corresponding gene, and the pathogen name to which vaccine was given. This figure was generated using Rawgraphs.

Overall, our search on “response to vaccine” phenotype at the GWAS catalog revealed various associations. The strongest genetic associations were linked to chromosome 6, particularly the HLA gene (Figure 2). Different associations, yet less significant, were found at different chromosomal locations, mapped to immune and non-immune related genes. Table 1 summarizes all the vaccine-related studies registered at the GWAS catalog, while the detailed list of reported SNPs is presented in Supplementary Table 1. Remarkably, most (around 65%) of the studies were conducted on the European or Asian populations. Moreover, the main trait for phenotypic classification was the antibodies or cytokines level after vaccine administration. In addition to the GWAS catalog, we used “Open Targets Genetics” portal to search for genetic associations with vaccine response. Figure 3 shows all the genes with an association score >0.11, along with the corresponding pathogen, while the details of the top 10 associations are summarized in Supplementary Table 2. This data again highlights the limitation in the currently available studies, as most of the significant associations are reported on few viruses only (smallpox, hepatitis B, measles, MMR, and rubella).

**Table 1:** List of all GWA studies on vaccine response retrieved from the GWAS catalog as of June 2021.

**Vaccine against**	**Phenotype**	**Study title**	**Trait**	**No. of associations**	**Discovery sample size and ancestry**	**References**
Rubella	Cellular immune response	Polymorphisms in the Wilms Tumor Gene Are Associated With Interindividual Variations in Rubella Virus-Specific Cellular Immunity After Measles-Mumps-Rubella II Vaccination.	Interferon-gamma secretion	0	1,643 European	(30)
			Interleukin-6 secretion	1	1,643 European 202 African American or Afro-Caribbean	
Hepatitis B	Antibody response	Key HLA-DRB1-DQB1 haplotypes and role of the BTNL2 gene for response to a hepatitis B vaccine.	Anti-HBV surface antigen IgG level	20	1,193 East Asian	(5)
Hepatitis B	Antibody response	GWAS identifying HLA-DPB1 gene variants associated with responsiveness to hepatitis B virus vaccination in Koreans	Anti-HBV surface antigen IgG level	1	6,867 East Asian	(25)
Measles-mumps-rubella	Cytokine production	Genome-wide SNP associations with rubella-specific cytokine responses in measles-mumps-rubella vaccine recipients.	IL-6 level	2	883 European	(4)
			IFN gamma level	8	883 European	
Measles	Neutralizing antibodies level	Genome-wide associations of CD46 and IFI44L genetic variants with neutralizing antibody response to measles vaccine.	IFN gamma level	1	2,555 European	(3)
			Neutralizing antibodies titer	6	317 African American or Afro-Caribbean	
Smallpox	Antibody response	Genome-wide association study of antibody response to smallpox vaccine.	IL-6 level	37	580 European 217 African American or Afro-Caribbean 217 Hispanic or Latin American	(31)
Smallpox	Cytokine production	Genome-wide analysis of polymorphisms associated with cytokine responses in smallpox vaccine recipients.	Secreted IFN-alpha level	32	512 European 199 African American or Afro-Caribbean	(32)
			Secreted IL-10 level	6		
			Secreted IL-12p40 level	10		
			Secreted IL-1beta level	13		
			Secreted IL-2 level	17		
			Secreted TNF-alpha level	6		
			Secreted IL-6 level	9		
Multiple vaccines	Antibody response	Common Genetic Variations Associated with the Persistence of Immunity following Childhood Immunization.	Haemophilus influenza type b polyribosylribitol phosphate IgG level	0	967 European	(33)
			Meningococcal C functional antibody titers	6	1,585 European	
			Meningococcal C IgG concentrations	1	1,203 European	
			Tetanus toxoid IgG concentrations	1	549 European	
Anthrax	Antibody response	A genome-wide association study of host genetic determinants of the antibody response to Anthrax Vaccine Adsorbed.	Anti-protective antigen (PA) ab	8	726 European	(7)
Hepatitis B	Antibody response	A genome-wide association study of hepatitis B vaccine response in an Indonesian population reveals multiple independent risk variants in the HLA region.	Anti HBs titer	3	1,683 Asian unspecified	(27)
Hepatitis B	Antibody response	A genome-wide association study identifies polymorphisms in the HLA-DR region associated with non-response to hepatitis B vaccination in Chinese Han populations.	Anti HBs titer	2	185 East Asian	(26)

Using genotype-phenotype association approach, several highly significant SNPs were reported. These polymorphisms are located in genes that are linked directly or indirectly to the immune response. For instance, variants in the interferon-induced protein 44 like (*IFI44L*) and the cluster of differentiation 46 (*CD46*) genes were associated with measles-specific neutralizing antibody titers in response to MMR vaccine (3). *IFI44L* encoded proteins are stimulated by interferon type 1 and hence, are possibly involved in the innate immune response (3). On the other hand, CD46 glycoprotein is involved in the regulation of complement and antibody-mediated lysis. Additionally, it is a cellular receptor for attenuated measles virus strains, group B and D adenoviruses, human herpesvirus 6, bovine viral diarrhea virus, and other pathogens (34). Interestingly, variants in these two genes have been previously associated with adverse events/febrile seizures following MMR vaccination (28). Additionally, genetic variants in *IFI44L* have shown to increase the susceptibility of mice to Coxsackievirus B3 virus, confirming the possible association of this gene to innate immunity (35). Other GWA studies identified genetic variants that could modulate the adaptive immune responses to MMR vaccinations. Kennedy et al. reported significant associations in the protein tyrosine phosphatase delta (*PTPRD*) and the iron regulatory protein (*ACO1*) genes, in response to MMR vaccine (4). These variants explained the inter-individual variations in IFNγ response to rubella virus stimulation. However, the exact role of these genes in vaccine-response still requires further explanation. Additionally, a variant in the Wilms Tumor Gene (*WT1*) has been linked to rubella-specific interleukin 6 secretion following MMRII vaccination (30). Although *WT1* gene is not typically associated with immunity, it has been shown that it can directly bind to IL-10 promoter and induce IL-10 expression, which is important for tumor necrosis factor-α- (TNF- α) induced IL-10 stimulation in macrophages (36).

In addition to MMR, smallpox vaccine is one the commonly studied vaccines in the context of host genetics. Multiple GWAs identified genetic variants in genes that modulated the humoral (neutralizing antibodies) or cellular (cytokine secretion) following vaccination (31, 32). More than 50 significant polymorphisms (reached the GWAS significance of 5 × 10^−8^) in different genes have been reported (Supplementary Table 1). These variants were found to affect the levels of anti-smallpox antibodies, IFN-alpha, IL-10, IL-12p40, TNF-alpha, and IL-6 (31, 32). Importantly, many if these variants are located genes that have never been linked to immunity. Hence, the pathways by which these variants affect antibody and cytokines production is largely unknown, and necessitates additional functional characterization.

Considering that 5–10% of individuals who receive hepatitis B virus (HBV) vaccine fail to produce protective antibodies, several GWASs were conducted to investigate the genetic factors behind this variability (5). The most significant associations were linked to *HLA* polymorphisms. Multiple *HLA* alleles were associated with anti-hepatitis surface antigen IgG levels including *HLA-DPB1* and *HLA-DRAB5* and *HLA-DQA1* (5, 25–27). *HLA* genes are known to be the most polymorphic region of human genes, and encodes surface proteins which are essential in self and non-self-antigen presentation (37). Therefore, it is expected that certain *HLA* haplotypes correlate to response to vaccination. Notably, *HLA* genes have been linked to the susceptibility or resistance of multiple infections, including HBV, SARS-CoV, and SARS-CoV-2 (38–40). In Addition to *HLA* types, significant associations were found in other genes such as the Butyrophilin Like 2 (*BTNL2*) gene, which is involved in the regulation of T cell activation (5, 41).

## Genetics and Vaccine Adverse Events

In the past decade, a new terminology, called “Adversomics” has been introduced by Whitaker et al. (42). This term refers to the study of vaccine-related adverse reactions using immunogenomics and systems biology approaches (42). Typically, the design of vaccines is based on stimulating the immune system to an antigen. This usually induces an inflammatory reaction, which ranges from a mild local to a serious systematic adverse reaction in rare cases. Indeed, vaccine adverse effects—whether real or unreal- have been one of the major barriers in public acceptance and trust in vaccines. Thus, the identification of factors that contribute to the unwanted vaccine adverse effects is crucial to increase the safety as well as to maintain public trust in vaccines.

It is well-acknowledged now that heterogeneity in vaccine response is a multifactorial trait influenced by external (environmental), and internal (host immunogenetics) factors. However, the field of adversomics is still relatively new compared to other fields and only a very few studies has been conducted so far (Table 2). Additionally, multiple studies that looked into the underlying genetic factors in individuals experiencing adverse effects did not report any GWAS significant associations (45). This could be attributed to the small sample sizes, which reflects the infrequency of serious adverse vaccines or the complexity of such analysis. On the other hand, few significant associations were found and replicated. For instance, Hallberg et al., reported a novel association between Pandemrix (influenza vaccine)-induced narcolepsy and the non-coding RNA gene (*GDNF-AS1*) (29). This gene is involved in regulating the expression of GDNF and have been linked to neurodegenerative diseases (29). Similarly, a GWAS identified significant risk variants for developing febrile seizures following MMR vaccine (28). These variants are located in *CD46* and *IFI44L* genes, and have also been linked to the humoral immune response to MMR vaccine as mentioned earlier. Rare variants have also played a major underlying factor in life-threatening disease following vaccinations with live-attenuated vaccines. For instance, inborn errors of IFN-γ, B-cell Immunity, IFN-α/β and IFN-λ, and adaptive immunity, were leading to Bacille Calmette-Guerin (BCG), oral poliovirus (OPV), vaccine measles virus (vMeV), and Oral rotavirus vaccine (ORV) diseases, respectively (49).

**Table 2:** List of all genome wide associaitons on vaccine adverse events.

**Vaccine against**	**Type of vaccine**	**Phenotype**	**Region**	**Main annotated gene**	**References**
SARS-CoV-2	mRNA vaccines: Pfizer/BioNTech (BNT162b1) and Moderna (mRNA-1273)	Vaccine-related adverse events: severe/extreme difficulties with daily routine	6p22.1	HLA-A*03:01	(43)
SARS-CoV-2	mRNA vaccines: Pfizer/BioNTech (BNT162b1) and Moderna (mRNA-1273)	Vaccine-related adverse events:	Multiple	Multiple genes including: *HLA, NOTCH4*, and *RPS18*	(44)
Influenza	Pandemrix	Vaccine-related adverse events: narcolepsy	5p13.2	*(GDNF) anti-sense 1 (AS1)*	(29)
Influenza	Intranasal trivalent live attenuated influenza vaccine (LAIV) intramuscular trivalent inactivated vaccine (TIV)	Vaccine-related adverse events: Wheezing	1q23.2	*CRP - AL445528.1*	(45)
		Vaccine-efficacy: Influenza infection	7p11.2	*LINC02854 - AC092848.2*	
Measles-mumps-rubella	Priorix or MMR II	Vaccine-related febrile seizures	1p31.1	*IFI44L*	(28)
			1q32.2	*CD46, CD34*	
Smallpox	Aventis Pasteur Smallpox vaccine	Fever, generalized rash, lymphadenopathy	1p36.3	*THFR*	(46)
			5q31.1	*IRF1*	
			5q31.1	*IRF1*	
Smallpox	Dryvax	Fever, acute Vaccinia syndrome	Multiple	*IL1, IL4*, and *IL18*	(47)
Yellow fever	YF-17D	Viscerotropic disease - Persistent viremia	Multiple	*CCR5* and its ligand *RANTES*	(48)
Yellow fever	YF-17D	Viscerotropic and Neurotropic disease	Multiple	*RANTES, IL-6, IL-8, MIG, GRO, MCP-1, TGF-β*, and *TNF-β*	(43)

Taken together, these studies, as well as others, reveal an important insight on the role of common and rare genetic variants in vaccine-related adverse events and underscore the need for more and larger studies.

## Immunogenomics and Vaccinomics of SARS-COV-2

### Immune Response to SARS-CoV-2 Infection

SARS-CoV-2 primarily attacks the respiratory system leading to pneumonia and lymphopenia in severe disease. However, in most cases, a 1-week, self-limiting respiratory disease occurs (50). Viral antigens, recognized by pathogen recognition receptors (PRRs), mainly TLR 3, 7, and 8, induce the enhanced production of IFNs. Similar to other coronaviruses, viral antigens trigger the development of antibody production, as well as CD4+ and CD8+ T-cells immunity.

Generally, SARS-CoV-2 infection leads to the production of anti-N and anti-S antibodies, with antibodies targeting the receptor-binding domain (RBD, in S1) being crucial for viral neutralization (51). Studies showed that most SARS-CoV-2 patients seroconvert, and neutralizing antibodies (nAbs) activity persists up to 6 months (51, 52). Interestingly, although there is an evidence of the beneficial role of nAb in protection against SARS-CoV-2, the peak-neutralizing activity was found to correlate positively with disease severity (52, 53). In fact, despite the numerous amount of studies in this field, there is still a knowledge gap in understanding the durability and effect of these antibodies on disease outcomes and re-infection.

A growing evidence highlights the important role of T-cell immunity in SARS-CoV-2, especially in patients with an underdeveloped humoral response. It was previously found that in contrast to anti- SARS-CoV-1 antibodies that wane after 2–3 years, T-cell responses are long lasting, and can be detected up to 17 years post recovery (54, 55). T-cells recognize viral peptides that are presented on the MHC class I (HLA in humans), which stimulates cytokine release and cytotoxic activity of CD8+ T cells (56). MHC class II can also present antigens to CD4+ T cells (56).

Importantly, as HLA system is known to be highly polymorphic, some haplotypes were found to influence individuals' susceptibility to many infections by modulating the immune response (37, 57). Certain polymorphisms at these loci encode for cell receptors that could lower the binding efficiency to some viral peptides and, therefore, blunt the immune system's normal defenses against the virus in vulnerable individuals (58).

### Heterogeneity in Response to SARS-CoV-2 Infection

Since the start of the current SARS-CoV-2 pandemic, scientists have been puzzling over the factors underlying the inter-individual and inter-population differences in COVID-19 clinical manifestations. Although the infection with SARS-CoV-2 principally attacks the respiratory system, it can also trigger a systematic immune reaction that leads to multiple organ failure. According to the reported data, SARS-CoV-2 can lead to extra-pulmonary diseases, including renal dysfunction, gastrointestinal complications, liver dysfunction, cardiac manifestations, mediastinal findings, neurological abnormalities, and hematological manifestations (59). Epidemiologists have identified age as the main factor for developing COVID-19 related complications, especially among patients over 65 years of age (60). On the other hand, younger individuals (<20 years) almost exclusively experienced another severe condition that has been linked to COVID-19, which is Multisystem Inflammatory Syndrome (MIS-C), that mimics Kawasaki disease (KD) (60). Importantly, this condition is believed to occur in genetically predisposed children following exposure to trigger such as viral infection (61, 62). Besides, black and Hispanic children showed an increase risk of developing MIS-C (63). Although this could be due to the increased burden of SARS-CoV-2 in the black and Hispanic populations, it does not rule out the possible role of population genetics in influencing SARS-CoV-2 related diseases.

Although inter-host clinical variability is the rule in the course of any human infection, the response to SARS-CoV-2 showed a great variability that was not explained by the commonly known factors such as age, sex, and comorbidities. While more than 80% experience mild/asymptomatic illness, 20% experience severe respiratory syndrome, which further progresses to critical illness requiring ventilation in 5% (64). Importantly, severe clinical presentation was observed even in young and previously healthy individuals (65). Hence, neither age nor the lack of comorbidity can guarantee a mild manifestation of the infection. In a study that investigated the transmission of SARS-CoV-2 among asymptomatic carriers, it was shown that family members who are living together tend to develop severe infection (66). This suggested the potential role of genetics in the manifestation of COVID-19.

The striking heterogeneity in the response to SARS-CoV-2 highlighted the crucial need to comprehend the underlying causes of interindividual differences, including host genetics. This area of research has expanded by the combined efforts of global consortiums as well as individual efforts. For instance, the COVID-19 Human Genetics Effort was rapidly launched at the beginning of this pandemic. Their aim was to identity monogenic errors of immunity that could lead to severe COVID-19 in young individuals who were previously well and developed life-threatening disease, such as pneumonia or MIC-S (67). On the contrary, the Host Genetics Initiative (HGI) was established to support the collection and sharing of GWAS data and results to understand the common variants contributing to susceptibility and severity to COVID-19 (68). These two groups, as well as others, have identified several genetic determinants that affect the response to SARS-CoV-2 infection.

The first case report that identified rare variants linked to COVID-19 applied rapid whole-exome sequencing approach on four young male patients (below 35 years) who had a severe SARS-CoV-2 infection. The study revealed rare putative loss-of-function variants of X-chromosomal *TLR7*, which resulted in impaired type I and II IFN responses (65). Additional following studies had also highlighted the role of variants related to IFN signaling in severe COVID-19. Using a larger sample size, Zhang et al. performed whole-genome or exome sequencing of 659 and 534 with life-threatening and mild SARS-CoV-2, respectively. Inborn errors of *TLR3*, interferon regulatory factor 7 (*IRF7*), and interferon regulatory factor 9 (*IRF9*) genes were investigated in life-threatening COVID-19 pneumonia patients who were previously healthy. These genes were selected as they were previously linked to critical influenza-associated pneumonia. The study identified rare variants predicted to be loss-of-function (LOF) related to TLR3- and IRF7-dependent type I IFN immunity in patients with severe SARS-CoV-2 infection (69). Notably, patients who had these mutations or had neutralizing autoantibodies to type I IFNs showed lower levels of IFNs, which possibly contributed to increased viral replication and pathogenesis (70).

On the other hand, the Host Genetics Initiative (HGI) provides the largest set of GWA studies and meta-analyses in history. The latest release (R6 – June 2021) included 125,584 SARS-CoV-2 cases and over 2.56 million controls. A total of 23 genome-wide significant loci (*P* < 5 × 10^−8^) were found to either associate with disease susceptibility (7 loci) or disease severity (16 loci). These variants were located in multiple genes related to viral entry, host immune response, lung function, and others. The severity lead variant was located in chromosome 3 (rs35508621), that is in LD with *LZTFL1* and has *CXCR6* as the highest gene prioritized by OpenTargetGenetics'V2G. The *LZTFL1* gene is involved in regulating protein trafficking to ciliary membranes and has a role in immune response, while *CXCR6* plays a role in chemokine signaling (71, 72). The most statistically significant variant on chromosome 1 was rs67579710, which was also associated with COVID-19 severity. This is an intronic variant in Thrombospondin 3 (*THBS3*) gene, which is related to lung function. Similarly, genetic variants in *SFTPD* (rs721917), *SLC22A31* (rs117169628), *FOXP4* (rs41435745), and *MUC5B* (rs35705950), which are all related to lung function and lung diseases, have been significantly associated with COVID-19 severity. *SFTPD* gene encodes the surfactant protein D (SP-D) that has a role in the innate immunity, while SLC22A31 belongs to the family of solute carrier proteins, and predicted to enable transmembrane transporter activity (73, 74). *FOXP4* is expressed in the proximal and distal airway epithelium and variants within this region have been linked to lung diseases (75, 76). MUC5B, on the other hand, produces a major gel-forming mucin in the lung which is important in mucociliary clearance (MCC) and host defense (77). *MUC5B* variant increases the expression of MUC5B in the lung, and therefore could provide a protective effect against SARS-CoV-2 progression (78). Furthermore, multiple other SNPs exhibited significant associations with severe COVID-19, including rs77534576 (*TAC4*), rs111837807 (*CCHCR1*), rs766826 (*ELF5)*, rs10774679 (*OAS1/OAS3/OAS2*), rs12809318 (*FBRSL1*), rs61667602 (*CRHR1*), rs2109069 (*DPP9*), rs11085727 (*TYK2*), rs1405655 (*NR1H2*), and rs13050728 (*IFNAR2*). Most of these genes have a role in the innate immune response, or lung inflammation. For instance, *TAC4* gene product has a role in blood pressure regulation, and in immune responses (72). *OAS* gene cluster, primarily *OAS3*, encodes for antiviral restriction enzyme activators that lead to degradation of viral ssRNA as a protective mechanism against viruses (79). Interestingly, the locus in OAS1/2/3 cluster, which has been associated with severe COVID-19 among individuals of European ancestry, has a protective haplotype of ~75 kilobases (kb) derived from Neanderthals (80). This haplotype was associated with a ~22% reduction in relative risk of becoming severely ill with COVID-19. *IFNAR2*, which encodes for interferon receptor, is critical for the antiviral host response. Mutation in the *IFNAR2* was reported to associate with critical illness in COVID-19 in a previous GWAS as well (81). *DPP9* and *TYK2*, on the other hand, are related to host-driven inflammatory lung injury, which is a main mechanism of late, life-threatening COVID-19 (81). Other genes, such as *ELF5* and *FBRSL1* have no previously reported lung trait associations, and therefore, will need further mechanistic characterization to understand their role in severe COVID-19.

In addition to severity, multiple variants were linked to susceptibility to SARS-CoV-2. A variant near *ACE2* gene (rs190509934) was significantly associated with acquiring SARS-CoV-2. On note, *ACE2* functionally interacts with *SLC6A20*, another gene that harbor a significantly associated SNP with SARS-CoV-2 susceptibility (rs73062389). Other significant SNPs were located near *NXPE3* gene on chromosome 3 (rs17412601), *PLEKHA4* on chromosome 19 (rs4801778), and *HLA-DPA1/HLA-DPB1* (rs2071351). These variants, along with the previously identified region in the *ABO* gene (at chromosome 9, rs505922), are likely modulating susceptibility to infection but not progression to a severe form (82, 83).

Besides the HGI, multiple GWA studies conducted by other consortia as well as independent research and genomics services groups identified SARS-CoV-2 related host genetic variants that influence SARS-CoV-2 outcomes, some of which were replicated in the HIG (78). It has been shown that genes related to renin-angiotensin-aldosterone system (RAAS); including the *ACE1* and *ACE2* gene polymorphisms, contribute to COVID-19 pathogenesis (84). Importantly, SARS-CoV-2 binding to the ACE2 receptor on cell surface requires cellular proteases that facilitate fusion between the virus membrane and the cell membrane, such as the TMPRSS2. Genetic polymorphisms in cellular proteases were suggested to affect SARS-CoV-2 susceptibility in various populations through *in silico* and *in vivo* studies (85, 86).

There is an accumulating evidence on the association of HLA with SARS-CoV-2 from various studies. However, many studies were unreproducible as they reported results of *in-silico* analysis, or were limited by small sample size and variability in participants' genetic ancestries. For instance, using *in-silico* analysis, it was reported that HLA-A^*^02:01 is associated with an increased risk of COVID-19. HLA-A^*^02:01 showed a relatively lower capacity to present SARS-CoV-2 antigens in comparision to other HLA class I molecules (87). In contrast, a later study that included 111 deceased COVID-19 patients and 428 volunteers reported that HLA-A^*^02:01, in addition to HLA-A^*^03:01 contributed to lower risk of severe COVID-19 (88). Another study conducted among 182 Sardinian SARS-CoV-2 patients suggested that the extended haplotype HLA-A^*^02:05, B^*^58:01, C^*^07:01, DRB1^*^03:01 has a protective effect against SARS-CoV-2 infection, in contrast to HLA-DRB1^*^08:01 allele which was associated with hospitalization (89). HLA-C^*^04:01 has been also suggested to correlate with severe clinical course of COVID-19 in a study on 435 patients from different countries (90). Additionally, a retrospective analysis on 265 Italian cohort showed that HLA-DRB1^*^08 was more frequent in SARS-CoV-2 infected patients, and correlated with mortality (91). Another small-size study on Italians (*n* = 99) reported that HLA-DRB1^*^15:01, -DQB1^*^06:02 and -B^*^27:07 were associated with severe COVID-19 (92). Despite highlighting the potential role of HLA genomics in COVID-19, these studies, as well as numerous others, necessitate validation and replication in larger cohorts. Notably, the latest findings of largest GWAS on SARS-CoV-2 by the HGI, reported multiple *HLA* related variants that associated with SARS-CoV-2 outcomes (73). Particularly, five variants (top SNP rs111837807) reached genome-wide statistical significance were located in the Coiled-Coil Alpha-Helical Rod Protein 1 (CCHCR1) gene, which is 110 kb downstream of HLA-C. These variants were associated with SARS-CoV-2 severity. Moreover, a variant within HLA-DPB1 3'UTR (rs2071351) was significantly associated with disease susceptibility (73).

A consistent feature of the SARS-CoV-2 pandemic is the male bias in disease severity (93). Remarkably, TMPRSS2 expression is regulated by the androgen receptor (AR) in non-prostatic tissues. This could be reason behind the high susceptibility of men to progress to severe COVID-19 (94). Delanghe et al. suggested that Y-chromosome haplogroup might influence SARS-CoV-2 outcomes, considering its role in immune and inflammatory responses (95). Nevertheless, the interaction between the AR, TMPRSS2, and Y-chromosome polymorphisms and their effect on COVID-19 outcomes is still not well-addressed.

In fact, any polymorphism located in genes related directly or indirectly to the host immune response could be associated with SARS-CoV-2 outcomes. Genetic variants in genes encoding the complement component 3 (C3), Interleukin-37, and vitamin D binding protein (DBP), were also suggested as factors influencing SARS-CoV-2 outcomes (96–98).

It is worth noting that host genetics studies did not only highlight the role of genetics in the inter-individual heterogeneity in response to SARS-CoV-2, but also added additional insights on the great differences in population genetics structure. For instance, a variant that was identified close to *FOXP4* and correlated with COVID-19 severity has a frequency that is largely variable between different populations. This variant is considered rare in Europeans, with a frequency of 1% in the population, compared to East-Asian (39%) and Hispanic/Latino (18%) populations (99). These results, as well as future genetic studies, could help in identifying the factors behind the inter-population differences in response to infections.

### Immune Response to SARS-CoV-2 Vaccines

Immediately after the release of SARS-CoV-2 genetic sequence, a race for developing a vaccine has started. Over 100 SARS-CoV-2 vaccines are at different stages of clinical development (100). Most of these vaccine candidates are based on the spike (S) protein, or part of it, considering its essential role in virus entry. Multiple platforms have been utilized in the vaccine design, including using non-replicating viral vectors, inactivated whole-virus, protein subunit, messenger RNA (mRNA), and DNA-based vaccines. At present, three vaccines (Pfizer-BioNTech BNT162b2, Moderna mRNA-1273, and Janssen Ad26.CoV2.S) had already received the emergency use authorization (EUA) from the U.S. Food and Drug Administration (FDA). Six other vaccine candidates are approved under EUA in different other countries (AstraZeneca, Novavax, CureVac, Sputnik V, Sinovac, Sinopharm) (101). Additionally, Pfizer, Moderna, Janssen, and AstraZeneca vaccines have received the European Medicines Agency (EMA) approval of use in the European Union, while multiple other vaccine candidates are still under EMA review (102).

Despite the fact that vaccines play a vital role in infection control and SARS-CoV-2 is no exception, the profound differences in response to SARS-CoV-2 raise the question of whether this clinical variability will also appear in response to vaccines. Importantly, different vaccine candidates induce different immune responses. Therefore, the response to vaccination could be modulated by distinct host immunogenetic determinants that are unique to that vaccine structure.

The two SARS-CoV-2 mRNA vaccines developed by Pfizer-BioNTech and Moderna were the first to enter the race, considering the speed of cloning and synthesis. These two vaccines were also the first to receive the approval for emergency use and are currently being widely distributed and administered (103). Both vaccines are lipid nanoparticle formulated nucleoside-modified mRNAs, encoding the pre-fusion SARS-CoV-2 full-length S protein with proline substitutions and produce combined adaptive humoral and cellular immune responses (51). Vaccination with BNT162b2 (Pfizer-BioNTech) elicits potent anti-S IgG antibodies after a single dose, and neutralizing antibodies at day 29 (7 days post-boost). Additionally, an S-specific CD8+ and T helper type 1 (Th1) CD4+ T cells response was observed in 91.9 and 94.1% respectively (104). Moreover, the expression of IFNγ and IL-2 and only minimal expression of IL-4 in BNT162b2-induced CD4+ T cells confirmed a Th1 response and the absence of the potentially harmful Th2 immune response (104). Similarly, Moderna mRNA-1273 vaccine elicited and immune response after the first dose that was boosted by the second injection. High titers of binding and neutralizing anti-S antibodies post-boost, which was accompanied with a dominant Th1 CD4+, but a minimal CD8+ T-cell response (105). From the clinical trials, Pfizer-BioNTech and Moderna-mRNA-1273 reported an overall vaccine efficacy of 94.1 and 94.6% respectively (101).

With a close but lower vaccine efficacy than mRNA vaccines, AstraZeneca and Johnson/Janssen vaccines were constructed utilizing adenoviral vectors that expresses the full-length SARS-CoV-2 spike protein. Given that there is pre-existing immunity to around 70 types of human adenoviruses, AstraZeneca (AZD1222) vaccine uses a chimpanzee-derived adenovirus (ChAdOx) to circumvent the concern of pre-existing immunity. This vaccine induced the production of neutralizing antibodies in 91% and 100% of participants after prime and boost doses, respectively. Moreover, T-cell immune response was induced, peaking at 14 days post-vaccination, as measured through IFN-γ enzyme-linked immunosorbent spot assay (106). Importantly, overall vaccine efficacy in preventing COVID-19 ranged between 62 and 90% as a result of multiple factors including the diverse ethnicity of the study population (107).

Similarly, Janssen vaccine (Ad26.COV2.S) was based on a recombinant, replication deficient adenovirus (Ad26) encoding a full-length and stabilized spike protein. This vaccine elicited humoral and cellular immune responses following a single dose. Neutralizing antibodies were detected in 90 and 100% of participants at days 29 and 57, respectively. Additionally, 76–83% of participants showed CD4+T-cell responses that induced the favorable polarized (Th1 over Th2) immune response. Moreover, CD8+ T-cell responses were detected in 51–64% of participants (108). The overall efficacy of the Ad26.CoV2.S vaccine was 72% in the US; 66% in Latin America, and 57% in South Africa (101).

Other vaccine candidates, which are either in-use or in different stages of clinical trials include inactivated vaccine derived from virus propagated in culture and then chemically inactivated. The inactivated virus expresses viral proteins that are conformationally native to the wild-type virus. Sinopharm and Sinovac are examples of SARS-CoV-2 inactivated vaccines produced in China. Despite the safety concerns related to such vaccines, including the risk of antibody-dependent enhancement, it was reported that these vaccines are safe and relatively efficient (Sinopharm: 79 and 86%—Sinovac: 78, 65, and 91.25% depending on dosing and population) (101). Nonetheless, several concerns have grown recently with regard to the real efficacy of these two vaccines. Countries where Sinovac and Sinopharm vaccines were used are still suffering from increase in COVID-19 cases, as recently reported from Mongolia, where half the people have received are vaccinated with Sinopharm (109).

Another vaccine platform that is currently used but classically has safety-related concerns is recombinant protein based vaccine. This type of vaccines has a potential risk of inducing the unfavorable Th2 biased immune response. However, this can be overcome with the use of appropriate adjuvants. Novavax vaccine (NVX-CoV2373) is an example of recombinant protein vaccine, which is composed of recombinant full-length, pre-fusion S protein with saponin-based Matrix-M adjuvant. The use of this adjuvant enhances the immune response and elicits high levels of neutralizing antibodies (110). The vaccine recorded an overall efficacy of 89.3% in UK and 60% in South Africa phase 3 clinical trials. Recently, the results of a larger clinical trial in the US and Mexico (involving almost 30,000 participants) showed an overall efficacy of 90.4% (111).

The immune response does not depend on the type of vaccine only (inactivated virus, mRNA, DNA, or protein subunit), but also on the type of adjuvant. Adjuvants are needed to activate the innate immune response through pattern recognition receptors (PRRs), which recognize pathogen-associate molecular patterns (PAMPs) (112). Depending on the type of vaccine, adjuvants can be endogenous or exogenous. Vaccines that are based on live-attenuated or killed whole virus usually contain an endogenous adjuvant that is sufficient to induce an adaptive immune response. Likewise, mRNA- and DNA-based vaccines contain an endogenous adjuvant which is the genomic material itself, yet, they require a lipid or polymer-based nanoparticles that acts as a protective vehicle to improve the vaccine uptake into cells (113). On the other hand, antigen based vaccines such as recombinant proteins require an adjuvant that acts as innate immune stimulator (114).

### Genetics and Response to SARS-CoV-2 Vaccines

Considering that SARS-CoV-2 vaccines are still new, studies on the immunogenetic determinants of vaccine efficacy are very limited. Theoretically, genetic polymorphisms in genes of the innate and adaptive immune system influence the individual response to vaccines, and SARS-CoV-2 vaccines are no exception. Actually, personalized approaches in SARS-CoV-2 vaccines are probably more important than in other vaccines, given the large inter-individual differences in response to SARS-CoV-2 infection. Analysis of host genetics factors contributing to SARS-CoV-2 clinical variability revealed a set of genetic variants that modulate response to infection. These variants could also contribute to vaccine responsiveness. For instance, a large-scale GWAS study has reported that a rare variant in the *ACE2* gene down-regulated ACE2 expression, and hence, reduces the risk of COVID-19 (115). Such variants could also modulate the response to vaccines that are based on live attenuated virus, if they depend on the interaction between ACE2 and SARS-CoV-2 spike protein. This hypothesis is not new, since genetic polymorphisms in genes coding two measles receptors, the signaling lymphocyte activation molecule (*SLAM*), and membrane cofactor protein (*CD46*), were reported to influence the immune response to live measles virus vaccination (21). These polymorphisms were hypothesized to modify measles virus binding, virus entry, or affect the level of receptor expression (116).

In addition to that, genetic mutations in genes related to pathogen sensing/recognition (e.g., TLRs), antigen presentation (e.g., HLA), and activation/maturation of lymphocytes could also affect vaccine efficacy. Multiple vaccine candidates use adjuvants as innate immune simulators, such as Novavax (protein subunit vaccine used with Matrix-M-adjuvant), Sinovac and Sinopharm vaccines (inactivated virus with aluminum hydroxide adjuvant), and BBV152 (inactivated virus with aluminum hydroxide gel adjuvant TLR7/8 agonist chemisorbed Algel) (117). These adjuvants could stimulate the activation of the pro-inflammatory NLRP3 pathway, or act as TLR7/8 agonists, bridging the innate and adaptive immune responses (118). Given the clear evidence of the genetics influencing response to vaccines to other viruses as we described above, it is of a great interest to explore whether variants in genes involved in antigen/adjuvants recognition and the subsequent immune response also contribute to SARS-CoV-2 vaccine success. Of note, rare variants in TLR3 and TLR7 have been already linked to COVID-19 in previous reports (65, 69). Therefore, they could influence response to vaccination as well.

In fact, despite the very promising data from clinical trials and real-word figures on SARS-CoV-2 vaccine efficacy, there are still a number of vaccine non-responders. Out of 52,280 hospital admissions in the UK during the second wave, 3,842 patients have received at least the first dose of a COVID-19 vaccination. This indicates that out of every 14 patients admitted to the hospital admission, one patients is at least partially vaccinated (119). Moreover, researchers reported 113 deaths among vaccinated individuals. Importantly, the majority of deaths occurred among the elderly group who were at risk of severe COVID-19. Additionally, most of the hospitalizations occurred in the 1–14 days post vaccination where immunity is not fully protective. However, there is still a number of hospitalized patients more than 21 days post-vaccination (120). This, indeed, requires further investigation to identify and understand the mechanism behind vaccine failure in this group, including the role of genetic factors.

Another critical area to explore is the effect of population genetics on SARS-CoV-2 vaccine efficacy. Notably, Black, Asian, and minority ethnic groups showed an increase in the risk of severe COVID-19 compared to other populations. Yet, despite being the most affected, these groups are relatively under-represented in vaccine trials published so far (121). Definitely, there have been great efforts to encourage the participation of these groups in vaccine clinical trials, but there is still smaller proportion of minority groups compared to other populations. For instance, out of the 552 participants in phase 2/3 Oxford–AstraZeneca trial (UK), only one participant (0.18%) was Black, and 19 (3.4%) were Asians. Moreover, the larger phase 3 interim results of the same vaccine (11,636 participants) indicated that only 0.1–0.7% and 10.4–11.1% of participants were Black in the UK and Brazil trials, respectively. Asians, on the other hand, represented 4.3–5.7% in the UK trial, and 2.6% in Brazil trial (107). Pfizer and Moderna randomized, controlled trials also indicated the underrepresentation of these groups. While more than 30,000 participants were included in each vaccine trial, Black and Asians represented 9.3 and 4.3% in Pfizer trial, compared to 10.2 and 4.6% in Moderna trial, respectively (121–123). Using machine-learning predictions, a study suggested that SARS-CoV-2 subunit peptides may not be robustly displayed by the MHC molecules in certain populations (124). SARS-CoV-2 vaccines developed by Moderna, Pfizer, AstraZeneca, and others, may not protect individuals of non-European genetic ancestries (such as Africans or Asians) at the same level of protection as in white people (58, 124). Given the significant role of population genetic structure in shaping the response to infection and vaccination, it is important to ensure the adequate inclusion of these populations in clinical trials as well as in immunogenomics and vaccinomics studies. Furthermore, it was reported that race and ethnicity information are missing from the data reported to the CDC during the 1st month of vaccination in the US (125). Indeed, collecting ethnicity information during vaccination is essential for population stratification to evaluate the vaccine efficacy accurately.

Immunogenetic factors may influence vaccine effectiveness and could contribute to vaccine adverse events as well. This has been evidenced from studies on influenza, MMR, smallpox, and yellow fever vaccines (28, 43, 45, 46, 48). Current data indicated minor side effects of mRNA and viral vector based vaccines, such as headache, fever, fatigue, and body aches. However, studies reporting serious side effects started to emerge, including vaccine-induced immune thrombotic thrombocytopenia and neurological disorders (126, 127). This is in fact not surprising, because as large populations become vaccinated, it is possible for rare side events to appear. Additionally, while most vaccine-related side effects would be expected to appear during the first few weeks to months after vaccination, long-term effects may also occur (103). Whether these serious side effects are associated with other underlying undiagnosed conditions or are resulting from certain genetic causes, this requires further investigation. Until now, there are only two studies that investigated the genetics of reactions to SARS-CoV-2 vaccines. The first GWAS included 17,440 participants who were queried about their reactions to SARS-CoV-2 vaccination (128). Results revealed a significant association of HLA-A*03:01 and chills, fever, fatigue, and generally feeling unwell. Of note, this association was statistically significant only for those who received the Pfizer-BioNTech vaccine, in comparison to Moderna vaccine which showed a smaller effect size. The second GWAS (in preprint) was conducted on 4,545 Japanese individuals and identified 14 associated loci with vaccine side effects (44). These loci, especially 6p21, were associated with the expression of many genes related to the immune response, including *HLA* genes, which were previously associated with SARS-CoV-2 outcomes. This study also revealed multiple associations with genes related to immunity, such as *NOTCH4* and *RPS18*. Of note, a variant in *NOTCH4* gene has been previously associated with critical illness in COVID-19 (81). These studies highlight again the importance of investigating the immunogenetic determinants of SARS-CoV-2 vaccine response in order to understand the factors shaping vaccine adverse reactions and effectiveness. Whether other host genetic variants that were associated with susceptibility or severity of SARS-CoV-2 are also effecting the response to immunization, this requires further research.

Previous reports showed the possible risk of serious vaccine adverse events in individuals with rare inborn errors of immunity (IEI), particularly with the administration of live attenuated viral vaccines. For example, live polio vaccine was linked to paralytic polio in patients with agammaglobulinaemia (129). Impaired IFN immunity has also been linked to severe illness following yellow fever or MMR vaccines in patients with IFNAR1, IFNAR2 or STAT1 and STAT2 deficiencies, respectively (130). Again, this raises the question of SARS-CoV-2 vaccine responsiveness in patients with IEI. Even if the risk of serious illness from live attenuated vaccine was reduced with the use of other vaccine platforms that have better safety (such as mRNA or protein subunit vaccines), still, these patients might not develop complete protection. In a recent study on the immunogenicity of SARS-CoV-2 vaccines on IEI patients, it was shown that vaccination on IEIs is safe, but immunogenicity is affected by specific therapies and genetic defects (131). In common variable immunodeficiency (CVID) patients, which is a condition that can be caused by genetic mutations in immune-related genes, the response to SARS-CoV-2 vaccines was different from response to infection (132). Vaccination with two doses of mRNA vaccine did not generate spike-specific memory B cells (MBCs), but atypical memory B cells (ATM) with low binding capacity to spike protein, in contrast to vaccination after natural SARS-CoV-2 infection, which generated spike-specific MBCs. Spike-specific T-cells responses were also induced in CVID patients with different rates (132). These studies highlight the importance of finding a suitable immunization strategy that ensures eliciting an adequate protection in patients with inborn errors of immunity, which could be different from strategy applied on healthy individuals. This might include the use of additional booster doses and combining different vaccines/adjuvants in order to produce broad immunity. Also, it is important to track patients with deficient humoral or cellular response to vaccine and investigate if there are any genetic errors responsible for their impaired immunity. Nonetheless, it is worth mentioning that the current use of advanced vaccine platforms and constructs, which are based on eliciting both humoral and cellular response, could help in inducing protective immunity in IEI patients, at least partially. Yet, additional studies are needed to evaluate the effectiveness of the current vaccines and estimate the durability of protection in individuals with different immunogenetic profiles.

## Future Prospective and Conclusion

Current findings underline the significant role of immunogenomics in SARS-CoV-2 clinical variability. Data from research on other viruses also provided insights on the impact of immunogenomics in vaccine response. Now, with multiple SARS-CoV-2 vaccines being administered around the world, we have to be prepared to address important questions such as 1- Are individuals with a genetic predisposition to severe COVID-19 also at risk of serious SARS-CoV-2 vaccine-related adverse events? 2- What are the factors contributing to the inter-individual and inter-population variability in vaccine response? 3- Are there variants linked to SARS-CoV-2 vaccine-induced antibody secretion as previously reported from other viruses? 4- Are there host genetic biomarkers that can be used to predict vaccine efficacy in the future? 5- Can heterologous prime boost doses offer immunological advantages in providing protection to multi-ethnic populations? While we do understand the challenges in addressing these questions, and more importantly, the difficulty in the translational implications of this area of research, we believe that in the future, we could have genetic markers identified as predictors of SARS-CoV-2 infection and vaccine response. Hopefully, these markers would guide health care providers in the process of selecting the best treatment, and probably the most suitable vaccine for an individual or a specific ethnic group.

## Author Contributions

HY and MS conceived and designed the study. MS wrote the first draft of the manuscript. HA and AA proofread and revised the manuscript. All authors contributed to the article and approved the submitted version.

## Funding

This work was supported by the Qatar University High Impact Grant (Grant Number: QUHI-BRC-20_21-1) and Student Grant (Grant Number: QUST-1-BRC-2022-399).

## Conflict of Interest

The authors declare that the research was conducted in the absence of any commercial or financial relationships that could be construed as a potential conflict of interest.

## Publisher's Note

All claims expressed in this article are solely those of the authors and do not necessarily represent those of their affiliated organizations, or those of the publisher, the editors and the reviewers. Any product that may be evaluated in this article, or claim that may be made by its manufacturer, is not guaranteed or endorsed by the publisher.
