# Supplementary material for: Will Host Genetics Affect the Response to SARS-CoV-2 Vaccines? Historical Precedents
Source: Front Med (Lausanne). 2022 Mar 11;9:802312. doi: 10.3389/fmed.2022.802312 (PMC8962369; doi:10.3389/fmed.2022.802312)
Supplement: Supplementary file 1 [file Data_Sheet_1.docx]

**Table S1.** A list of SNPs that were reported to influence vaccine response retrieved from the GWAS catalog

| **Vaccine** | **Study population** | **Phenotype** | **Region** | **Gene** | **SNPS** | **P-VALUE** | **Reference** |
| --- | --- | --- | --- | --- | --- | --- | --- |
| Smallpox vaccine | 217 African American 580 Europeans 217 Hispanics | Neutralizing antibodies to Smallpox antigen | 10p12.1 | *MKX* | rs10508727 | 1.00E-10 | Ovsyannikova et al., 2012 |
|  |  |  | 8p12 | *LOC388460* | rs10503951 | 3.00E-09 |  |
|  |  |  | 10p12.1 | *GPR158* | rs12775535 | 4.00E-09 |  |
|  |  |  | 8q24.13 | *ZHX2* | rs10108684 | 1.00E-08 |  |
|  |  |  | 18p11.21 | *SPIRE1* | rs9959145 | 3.00E-08 |  |
|  |  |  | 1q43 | *GREM2* | rs10495471 | 2.00E-07 |  |
|  |  |  | 8p23.2 | *CSMD1* | rs17070309 | 5.00E-07 |  |
|  |  |  | 21q22.12 | *RUNX1* | rs2834655 | 5.00E-07 |  |
|  |  |  | 1q31.1 | *LOC647132* | rs10489759 | 8.00E-08 |  |
|  |  |  | 10q21.1 | *PCDH15* | rs12256830 | 2.00E-10 |  |
|  |  |  | 10p15.1 | *PRKCQ* | rs4748153 | 3.00E-08 |  |
|  |  |  | 6p21.2 | *LOC100131899, KIF6* | rs9380880 | 1.00E-07 |  |
|  |  |  | 10q23.33 | *CYP2C9* | rs2860975 | 2.00E-07 |  |
|  |  |  | 12q24.33 | *ANKLE2, GOLGA3* | rs12282 | 3.00E-07 |  |
|  |  |  | 10q23.33 | *CYP2C8* | rs1934953 | 5.00E-07 |  |
| Measles vaccine | 2,555 European & 317 African Americans | Measles specific neutralizing antibodies level | 1p31.1 | *IFI44L* | rs1333973 | 1.00E-10 | Haralambieva, et al., 2018 |
|  |  |  | 1q32.2 | *CD46* | rs2724384 | 3.00E-09 |  |
|  |  |  | 1q32.2 | *CD46* | rs2724374 | 5.00E-09 |  |
|  |  |  | 1p31.1 | *IFI44L* | rs273259 | 2.62E-08 |  |
| Smallpox vaccine | 512 European  199 African American | Cytokine level (secreted IFN-alpha) | 2p14 | *WDR92* | rs4078978 | 2.00E-18 | Kennedy RB, et al., 2012 |
|  |  |  | 5q31.2 | *ECSM2, TMEM173* | rs13181561 | 3.00E-14 |  |
|  |  |  | Xq27.3 | *LOC100128265* | rs381365 | 2.00E-12 |  |
|  |  |  | 4q31.22 | *ZNF827* | rs2048161 | 6.00E-12 |  |
|  |  |  | 14q23.1 | *MNAT1* | rs6573333 | 3.00E-10 |  |
|  |  |  | 12q13.3 | *TIMELESS* | rs11171846 | 6.00E-10 |  |
|  |  |  | Xq28 | *MAMLD1* | rs17252936 | 6.00E-10 |  |
|  |  |  | 1p13.2 | *KCND3* | rs12044963 | 9.00E-10 |  |
|  |  |  | 3q25.2 | *LOC152118* | rs6778194 | 2.00E-09 |  |
|  |  |  | 2q36.3 | *COL4A4* | rs2272205 | 3.00E-09 |  |
|  |  |  | 9q33.2 | *RAB14* | rs9408928 | 4.00E-09 |  |
|  |  |  | 15q12 | *ATP10A* | rs6576443 | 6.00E-09 |  |
|  |  |  | Xp22.11 | *PHEX* | rs1540283 | 9.00E-09 |  |
|  |  |  | 2q14.3 | *LOC728241* | rs17007761 | 1.00E-08 |  |
|  |  |  | 6p22.1 | *OR2H1* | rs4713226 | 2.00E-08 |  |
|  |  |  | 2q23.3 | *LOC344332* | rs10195263 | 3.00E-08 |  |
|  |  |  | 9q33.2 | *CEP110* | rs9408926 | 4.00E-08 |  |
|  |  |  | 14q22.2 | *BMP4* | rs210359 | 8.00E-08 |  |
|  |  |  | 3q28 | *LPP* | rs13067593 | 8.00E-08 |  |
|  |  |  | Xp22.11 | *PTCHD1* | rs5925760 | 1.00E-07 |  |
|  |  |  | 10p15.3 | *DIP2C* | rs17221323 | 1.00E-07 |  |
|  |  |  | 2p22.3 | *LTBP1* | rs542631 | 1.00E-07 |  |
|  |  |  | Xq25 | *ODZ1* | rs7060947 | 1.00E-07 |  |
|  |  |  | 1p13.3 | *CYMP* | rs4839431 | 2.00E-07 |  |
|  |  |  | 4p13 | *ATP8A1* | rs10517025 | 2.00E-07 |  |
|  |  |  | 21q22.3 | *LOC100129027* | rs8127571 | 2.00E-07 |  |
|  |  |  | 18q21.2 | *ELAC1* | rs9807334 | 2.00E-07 |  |
|  |  |  | 5q31.2 | *UBE2D2* | rs261532 | 3.00E-07 |  |
|  |  |  | 13q22.1 | *hCG_1820717* | rs17714988 | 3.00E-07 |  |
|  |  |  | 6q23.2 | *SGK1* | rs9493873 | 4.00E-07 |  |
|  |  |  | 9q21.33 | *DAPK1* | rs3095748 | 5.00E-07 |  |
|  |  |  | 19q13.43 | *NLRP13, NLRP8* | rs2043599 | 5.00E-07 |  |
|  |  | Secreted IL-10 | 4q32.3 | *SPOCK3* | rs13111850 | 3.00E-11 |  |
|  |  |  | 7q21.3 | *DYNC1I1* | rs13231718 | 5.00E-09 |  |
|  |  |  | 7q33 | *LOC100129730* | rs17231212 | 2.00E-08 |  |
|  |  |  | 16p12.3 | *DNAH3* | rs16970881 | 1.00E-07 |  |
|  |  |  | 5q14.3 | *LOC100131236* | rs10055544 | 1.00E-07 |  |
|  |  |  | 1p32.2 | *DAB1* | rs6679454 | 3.00E-07 |  |
|  |  | Secreted IL-12p40 | 6p22.3 | *LOC100131805* | rs7771911 | 2.00E-12 |  |
|  |  |  | 7q31.31 | *KCND2* | rs17142462 | 3.00E-09 |  |
|  |  |  | 11q23.1 | *BCO2* | rs7105056 | 1.00E-08 |  |
|  |  |  | 4q31.23 | *ARHGAP10* | rs7658486 | 1.00E-08 |  |
|  |  |  | 5q31.2 | *GFRA3* | rs11242417 | 6.00E-08 |  |
|  |  |  | 5q23.1 | *PRR16* | rs1584468 | 9.00E-08 |  |
|  |  |  | 11p12 | *LOC100132631* | rs11034653 | 2.00E-07 |  |
|  |  |  | 7q21.3 | *COL1A2* | rs3736638 | 3.00E-07 |  |
|  |  |  | 17p12 | *ADORA2B* | rs859267 | 3.00E-07 |  |
|  |  |  | 7q11.23 | *FKBP6, TRIM50* | rs6943090 | 3.00E-07 |  |
|  |  | Secreted IL-1beta | 10q22.1 | *SUPV3L1, VPS26A* | rs10998624 | 1.00E-09 |  |
|  |  |  | 10p14 | *LOC389936* | rs12247397 | 3.00E-09 |  |
|  |  |  | 22q13.33 | *FLJ44385* | rs17000918 | 4.00E-09 |  |
|  |  |  | 4q13.3 | *EPGN, MTHFD2L* | rs16850885 | 7.00E-09 |  |
|  |  |  | 4q13.3 | *MTHFD2L* | rs16850864 | 7.00E-09 |  |
|  |  |  | 7p15.2 | *LOC392008* | rs11564024 | 3.00E-08 |  |
|  |  |  | 13q32.3 | *SLC15A1* | rs9582259 | 3.00E-08 |  |
|  |  |  | 7p21.3 | *COL28A1* | rs17168526 | 6.00E-08 |  |
|  |  |  | 3q22.1 | *RAB6B* | rs9835973 | 7.00E-08 |  |
|  |  |  | 8q12.1 | *XKR4* | rs12542677 | 2.00E-07 |  |
|  |  |  | 4q31.3 | *DCHS2* | rs902464 | 3.00E-07 |  |
|  |  |  | Xq21.33 | *LOC100128151* | rs4827947 | 3.00E-07 |  |
|  |  |  | 3q26.2 | *MDS1* | rs9883650 | 4.00E-07 |  |
|  |  | Secreted IL-2 | 6q23.3 | *GAPDHL19* | rs9389316 | 7.00E-13 |  |
|  |  |  | 3p21.1 | *RFT1* | rs13088281 | 3.00E-11 |  |
|  |  |  | 1q42.3 | *TOMM20* | rs908327 | 5.00E-10 |  |
|  |  |  | 3p21.1 | *ITIH3, ITIH4* | rs17331151 | 2.00E-09 |  |
|  |  |  | 11q22.1 | *CNTN5* | rs11223581 | 9.00E-09 |  |
|  |  |  | 12p13.1 | *GRIN2B* | rs2268118 | 1.00E-08 |  |
|  |  |  | 17q21.33 | *NGFR* | rs16948200 | 2.00E-08 |  |
|  |  |  | 7q11.22 | *AUTS2* | rs1403155 | 2.00E-08 |  |
|  |  |  | 2q23.2 | *LOC100129594* | rs10432496 | 6.00E-08 |  |
|  |  |  | 14q21.3 | *ATP5GP2* | rs11845208 | 1.00E-07 |  |
|  |  |  | 11q12.2 | *DAGLA* | rs4963243 | 2.00E-07 |  |
|  |  |  | 6q27 | *FRMD1* | rs1473500 | 3.00E-07 |  |
|  |  |  | 3q25.2 | *P2RY1* | rs10513432 | 4.00E-07 |  |
|  |  |  | 13q33.2 | *LOC341604* | rs1372791 | 4.00E-07 |  |
|  |  |  | 6q22.31 | *LOC728727* | rs1392089 | 4.00E-07 |  |
|  |  |  | 17q23.2 | *BCAS3* | rs7224438 | 4.00E-07 |  |
|  |  |  | 3p21.1 | *TMEM110* | rs3796352 | 5.00E-07 |  |
|  |  | Secreted TNF-alpha | 12q12 | *PUS7L, IRAK4* | rs4251424 | 2.00E-08 |  |
|  |  |  | 2p21 | *CAMKMT* | rs13414205 | 7.00E-08 |  |
|  |  |  | 22q12.3 | *LOC441996* | rs738968 | 8.00E-08 |  |
|  |  |  | 3p21.31 | *SLC6A20* | rs758386 | 2.00E-07 |  |
|  |  |  | 2q36.3 | *C2orf83, LOC729968* | rs11889798 | 4.00E-07 |  |
|  |  |  | 2p13.1 | *SLC4A5* | rs13006863 | 5.00E-07 |  |
|  |  | IL-6 | 18q21.2 | *MEX3C* | rs8096445 | 9.00E-09 |  |
|  |  |  | 5q11.2 | *PDE4D* | rs17444059 | 2.00E-08 |  |
|  |  |  | 2p22.3 | *LTBP1* | rs6728021 | 4.00E-08 |  |
|  |  |  | 3q28 | *LOC100129725* | rs1516489 | 8.00E-08 |  |
|  |  |  | 9p21.1 | *NDUFB6, TOPORS* | rs17290760 | 1.00E-07 |  |
|  |  |  | 6q22.33 | *C6orf190* | rs17299841 | 2.00E-07 |  |
|  |  |  | 1p36.12 | *CDC42* | rs2501276 | 2.00E-07 |  |
|  |  |  | 8p23.1 | *BLK* | rs2255327 | 3.00E-07 |  |
|  |  |  | 5q34 | *ODZ2* | rs2973662 | 5.00E-07 |  |
| Rubella vaccine | 202 African American or Afro-Caribbean  1643 European | Interleukin-6 secretion | 11p13 | *WT1* | rs4986811 | 5.00E10-8 | Voigt EA, et al., 2017 |
| Hepatitis vaccine | 1193 East Asian | Anti-HBV surface antigen IgG level | 6p21.32 | *OXA1L* | rs3132969 | 2.00E-06 | Nishida N et al., 2018 |
|  |  |  | 4q32.3 | *TLL1* | rs17588573 | 6.00E-06 |  |
|  |  |  | 6p21.33 | *DDR1* | rs10947096 | 2.00E-06 |  |
|  |  |  | 6p21.33 | *C6orf15* | rs12527394 | 5.00E-08 |  |
|  |  |  | 6p21.33 | *HLA-C* | rs3130473 | 8.00E-06 |  |
|  |  |  | 6p21.32 | *BTNL2* | rs4248166 | 1.00E-12 |  |
|  |  |  | 6p12.2 | *TMEM14A* | rs6923338 | 8.00E-06 |  |
|  |  |  | 18q12.1 | *CDH2* | rs4355011 | 7.00E-06 |  |
|  |  |  | 6p21.32 | *HLA-DRA* | rs2395179 | 5.00E-14 |  |
|  |  |  | 6p21.32 | *HLA-DQA1* | rs34039593 | 6.00E-12 |  |
|  |  |  | 6p21.32 | *HLA-DQA2* | rs7745040 | 2.00E-10 |  |
|  |  |  | 6p21.32 | *TAP2* | rs1015166 | 1.00E-08 |  |
|  |  |  | 6p21.32 | *HLA-DPB2* | rs9277549 | 6.00E-11 |  |
|  |  |  | 6p21.33 | *PSORS1C1* | rs3819686 | 7.00E-06 |  |
|  |  |  | 6p21.32 | *C6orf10* | rs9268202 | 8.00E-08 |  |
|  |  |  | 6p21.32 | *HLA-DRAB5* | rs9268831 | 1.00E-13 |  |
|  |  |  | 6p21.32 | *HLA-DQB1* | rs9273062 | 2.00E-10 |  |
|  |  |  | 6p21.32 | *HLA-DPA1* | rs9277176 | 1.00E-07 |  |
|  |  |  | 6p21.32 | *HLA-DPB1* | rs9277464 | 7.00E-11 |  |
|  |  |  | 22q11.21 | *MICAL3* | rs5747408 | 9.00E-06 |  |
| Hepatitis vaccine | 6867 East Asian | Anti-HBV surface antigen IgG level | Hepatitis B | *HLA-DPB1* | rs9277356 | 7.00E-31 | Chung S et al., 2019 |
| Measles-mumps-rubella vaccine | 883 European | (IL-6 response) | 17p12 | *DNAH9* | rs7218761 | 1.00E-07 | Kennedy et al., 2014 |
|  |  |  | 2p22.1 | *LOC400950, SLC8A1* | rs4140752 | 8.00E-07 |  |
|  |  | (IFN gamma response) | 13q12.13 | *CDK8* | rs3736995 | 3.00E-07 |  |
|  |  |  | 16q23.1 | *MON1B, CNTNAP4* | rs1982955 | 4.00E-07 |  |
|  |  |  | 9p21.1 | *LOC100288436, ACO1* | rs10813374 | 3.00E-08 |  |
|  |  |  | 15q26.2 | *LOC145820, NR2F2* | rs9806400 | 2.00E-07 |  |
|  |  |  | 20p13 | *STK35, PDYN* | rs6112821 | 3.00E-07 |  |
|  |  |  | 9p24.1 | *PTPRD* | rs16928280 | 3.00E-10 |  |
|  |  |  | 14q11.2 | *RNASE9, RNASE10* | rs1243704 | 7.00E-07 |  |
|  |  |  | 2q36.1 | *EPHA4, SLC4A3* | rs1430246 | 2.00E-07 |  |
| Meningococcal C vaccine | 1585 European | Anti-meningococcal C serum bactericidal antibody measurement | 20p13 | *LOC105369219* | rs6135736 | 6.00E-08 | O'Connor et al., 2019 |
|  |  |  | 20p13 | *intergenic* | rs4814511 | 4.00E-07 |  |
|  |  |  | 20p13 | *SIRPG* | rs3818168 | 6.00E-07 |  |
|  |  |  | 20p13 | *SIRPG-AS1, SIRPG* | rs17770331 | 7.00E-07 |  |
|  |  |  | 4q13.1 | *AC097655.1 - LINC02496* | rs10517505 | 3.00E-06 |  |
|  |  |  | 20p13 | *SIRPG-AS1, SIRPG* | rs4813191 | 8.00E-06 |  |
|  | 1203 European | Meningococcal C IgG concentrations | 4p16.3 | *GAK* | rs3755963 | 9.00E-07 |  |
| Tetanus toxoid vaccine | 549 European | Tetanus toxoid IgG concentrations | 6p21.33 | *AL645933.4* | rs2523650 | 1.00E-09 |  |
| Anthrax vaccine | 726 European | Anti protective antigen (PA) ab | 18q21.2 | *SMAD4, MEX3C* | rs7230711 | 1.00E-06 | Pajewski et al., 2012 |
|  |  |  | 1p36.22 | *SPSB1* | rs11121382 | 4.00E-06 |  |
|  |  |  | 5q31.1 | *H2AFY, PITX1* | rs634308 | 4.00E-06 |  |
|  |  |  | 9q33.1 | *ASTN2* | rs6478282 | 6.00E-06 |  |
|  |  |  | 6p21.32 | *HLA-DRB1, HLA-DQA1* | rs3104402 | 6.00E-06 |  |
|  |  |  | 13q14.3 | *OLFM4, PCDH8* | rs732949 | 8.00E-06 |  |
|  |  |  | 9p21.1 | *TAF1L, TMEM215* | rs10758161 | 8.00E-06 |  |
|  |  |  | 4q24 | *TET2, PPA2* | rs2647264 | 9.00E-06 |  |
| Hepatitis vaccine | 1683 Asian | anti-HBs titer | 6p21.32 | *HLA-DR* | rs3135363 | 7.00E-22 | Png et al., 2011 |
|  |  |  | 6p21.32 | *HLA-DPB1* | rs9277535 | 3.00E-12 |  |
|  |  |  | 6p21.33 | *HLA* | rs9267665 | 1.00E-17 |  |
| Hepatitis vaccine | 185 East Asian | anti-HBs titer | 6p21.32 | *HLA-DRA, BTNL2, HLA-DRB1, C6orf10* | rs477515 | 3.00E-19 | Pan et al., 2013 |
|  |  |  | 6p21.32 | *HLA-DR* | rs3135363 | 8.00E-07 |  |
| Influenza vaccine | 67 European | Vaccine-related adverse events: Wheezing | 1q23.2 | *CRP - AL445528.1* | rs11265263 | 3.00E-06 | Miller et.al., 2011 |
|  |  | Vaccine-efficacy: Influenza infection | 7p11.2 | *LINC02854 - AC092848.2* | rs6593122 | 4.00E-06 |  |
| Influenza vaccine | 41 European, 1 other | Vaccine-related adverse events: narcolepsy | 5 | *(GDNF) anti-sense 1 (AS1)* | rs62360233 | 2.60E-09 | Hallberg et al., 2019 |
| Measles-mumps-rubella vaccine | 929 European | vaccine-related febrile seizures | 1p31.1 | *IFI44L* | rs273259 | 6.00E-12 | Feenstra et al., 2014 |
|  |  |  | 1q32.2 | *CD46, CD34* | rs1318653 | 1.00E-10 |  |

**Table S2.** Top genes with the highest association score in response to vaccine, retrieved from Open Target genetics platform.

| **Gene symbol** | **Full name** | **Association score** | **Reported vaccine** | **Possible/ reported function in immunity*** |
| --- | --- | --- | --- | --- |
| *IFI44L* | Interferon-induced protein 44 like | 0.384 | Measles | - Stimulated by interferon type I and thus is likely to be involved in innate immunity. - Important antiviral effector of type I interferon response against hepatitis C virus, IAV and RSV. - Negatively modulates the antiviral state induced by an analog of double-stranded RNA (dsRNA) or by IFN treatment. |
| *CD46* | CD46 molecule | 0.347 | Measles | - Regulator of complement activation (protecting cells from complement and antibody-mediated lysis). - Cellular receptor for measles virus attenuated (vaccine) strains, as well as for other pathogens (group B and D adenoviruses, human herpesvirus 6, bovine viral diarrhea virus, pathogenic Neisseria and Streptococcus pyogenes |
| *SLC15A1* | Solute carrier family 15 member 1 | 0.331 | Smallpox | ND |
| *PRKCQ* | Protein kinase C theta | 0.325 | Smallpox | - T cell activation and IL-4 regulation properties |
| *WT1* | WT1 transcription factor | 0.320 | Rubella | - Binds directly IL-10 promoter and induces IL-10 expression - Necessary for tumor necrosis factor-α–induced interleukin 10 (IL-10) stimulation in macrophages |
| *MKX* | Mohawk homeobox | 0.317 | Smallpox | - Interferon stimulated gene - Might have a role in innate immunity - Regulator of collagen expression and tendon differentiation |
| *ZNF827* | Zinc finger protein 827 | 0.295 | Smallpox | - Involved in transcriptional regulation and immune responses. |
| *GRIN2B* | Glutamate ionotropic receptor NMDA type subunit 2B | 0.295 | Smallpox | - ND |
| *HLA-DRA* | Major histocompatibility complex, class II, DR alpha | 0.294 | Hepatitis B | - Displays antigenic peptides on antigen presenting cells (APCs) for recognition by alpha-beta T cell receptor (TCR) on HLA-DR-restricted CD4-positive T cells. - Guides antigen-specific T-helper effector functions, both antibody-mediated immune response and macrophage activation. |
| *ARHGAP10* | Rho GTPase activating protein 10 | 0.293 | Smallpox | - Regulates Rho-GTPase, which are involved in signaling of T lymphocytes. |

*****ND: No data
